# Epidemiological Features of Infectious Diseases in Children and Adolescents: A Population-Based Observational Study in Shandong Province, China, 2013–2017

**DOI:** 10.3390/children11030309

**Published:** 2024-03-05

**Authors:** Wenjing Wang, Haitao Wang, Ke Song, Baoyu Wang, Fuzhong Xue, Lin Zhao, Wuchun Cao, Cheeloo EcoHealth Consortium (CLEC)

**Affiliations:** 1 Institute of EcoHealth, School of Public Health, Cheeloo College of Medicine, Shandong University, Jinan 250102, China; wangwenjing1996@mail.sdu.edu.cn (W.W.); wanghaitao@sdu.edu.cn (H.W.); 201915791@mail.sdu.edu.cn (K.S.); 202036459@mail.sdu.edu.cn (B.W.); zhaolin1989@sdu.edu.cn (L.Z.); 2 Department of Biostatistics, School of Public Health, Cheeloo College of Medicine, Shandong University, Jinan 250102, China; xuefzh@sdu.edu.cn; 3 Department of Epidemiology, School of Public Health, Cheeloo College of Medicine, Shandong University, Jinan 250102, China; 4 State Key Laboratory of Pathogen and Biosecurity, Beijing Institute of Microbiology and Epidemiology, Beijing 100071, China

**Keywords:** infectious diseases, children and adolescents, epidemiology, big data

## Abstract

Background: The arrival of the big-data era provides us with a chance to elaborate the spectrum and epidemiological characteristics of infectious diseases in children and adolescents aged 0–18 years in the pre-COVID-19 pandemic era. Methods: We collected data on infectious diseases in 891,981 participants from the Cheeloo Lifespan Electronic Health Research Data-library. The incidence density of each infection was calculated and stratified by age and region. The annual percentage change (APC) in incidence was estimated by logarithmic linear regression. Results: A total of 18,183 cases of 78 infections were diagnosed, with an overall incidence density of 626.33 per 100,000 person-years (PY). Of these, 6825 cases of 50 non-notifiable infectious diseases were identified. Children aged 1–3 years had the highest incidence of infections. The overall incidence revealed a significant increasing trend from 2013 to 2017 (APC = 36.9%, *p* < 0.05). Hand, foot, and mouth disease, pneumonia, and influenza were the three most common diseases. The incidence of pneumonia, rubella, scarlet fever, zoster, molluscum contagiosum, and syphilis increased significantly during the study period (all *p* < 0.05). Taian, Binzhou, and Weihai had the highest incidence of all other cities. The incidence of gastrointestinal infections increased markedly in the eastern coastal regions. Conclusions: More stress should be placed on a number of non-notifiable infectious diseases with a high burden and a significant increasing trend. Age-based and regional targeting efforts are needed to prevent and contain infectious diseases among children and adolescents.

## 1. Introduction

Infectious diseases constitute a great menace to humans worldwide. They are one of the greatest problems facing children today [1,2,3]. After China adopted the selective two-child policy in 2013, the number of births increased in the immediate period [4]. By the end of 2017, the number of people aged 0–19 years had reached 305 million in China [5]. Children and adolescents are prone to most infectious diseases due to their anatomical and physiological characteristics, immature immune systems, and habits of learning and playing in crowded places, which makes them a priority for public health policy in China [6,7,8]. 

According to the global estimates of cause-specific mortality in 2013, of the 6.3 million children who died before age 5 years, 51.80% (3.26 million) died of infectious causes, with pneumonia, diarrhea, and malaria being the leading infectious causes [9]. Among the 1.42 million patients with community-acquired pneumonia in 23 provinces of urban China in 2016, 34.83% of the patients were aged < 18 years [10]. Children under 5 years had the highest incidence rate of pneumonia [11]. Between 2005 and 2013, 85% (2763) of influenza outbreaks occurred in primary and middle schools in China, with an average of 30–99 students involved per outbreak [12]. Furthermore, epidemics of emerging infectious diseases, such as coronavirus disease (COVID-19) and influenza A H1N1 [13,14,15], and the resurgence of traditional infectious diseases, such as scarlet fever, undermined the health of children and adolescents [16]. Especially with the emergence of the COVID-19 pandemic, it is likely that the epidemiology of pediatric infectious diseases may change in the post-pandemic era. Therefore, the surveillance and investigation of the epidemiologic characteristics of pediatric infectious diseases at the population level pre-COVID-19 pandemic are urgently needed. 

The previous studies concerning infectious diseases in children and adolescents were mainly based on data from the China Information System for Disease Control and Prevention (CISDCP), covering 40 national notifiable infectious diseases [8,17]. Cases with other infections may not be reported and managed in time. With the advent of medical big data, a much broader range of data are available with infectious disease surveillance. In the present study, we collected data from the Cheeloo Lifespan Electronic Health Research Data-library (Cheeloo LEAD) to understand the spectrum and epidemiological characteristics of infectious diseases in children and adolescents in Shandong Province, China, and to guide targeted public health advice for early intervention and timely control of infectious diseases.

## 2. Materials and Methods

### 2.1. Data Source and Study Population

We performed an observational population-based study from 1 January 2013 to 30 June 2017 in Shandong Province, which was based on the Cheeloo LEAD. The Data library was established by the Health Commission of Shandong Province in 2017 [18] and connects multiple health-related sources involving electronic health records (EHRs), electronic medical records (EMRs), resident medical insurance payment systems and death registries by means of a common resident identity card number (Figure S1). Therefore, it enables medical information to be connected between different healthcare institutions, so we can obtain the medical history information of participants, such as disease diagnosis and symptoms. 

Multi-stage sampling was used to select the 4,688,388 potential study participants from rural and urban areas of Shandong Province, China. First, we conducted two simple random samplings, which yielded a number of communities in each district and towns in each country. Then, community residents (*n* = 1,375,494) and village residents (*n* = 1,873,911) were obtained by cluster sampling in these communities and towns, respectively. Third, by second cluster sampling from the local EHRs and EMRs, pregnant and postpartum women (*n* = 249,569), primary and middle school students (*n* = 471,666), infants and young children (*n* = 201,116), and the working population (*n* = 516,632) were obtained. The sampling procedure is shown in Figure S1. The sample population of each city was more than 150,000, accounting for about 2–13% of the local population [19]. Participants with at least one record in the platform from 1 January 2013 to 30 June 2017 were included, and those with duplicate or incorrect identity card numbers, insufficient baseline information and aged over 18 years were excluded. Then, a total of 891,981 participants aged 0–18 years were enrolled.

Our study was approved by the Ethics Committee of the School of Public Health, Shandong University. Researchers must obtain approval from an official review board before they use encrypted data on the Cheeloo LEAD’s servers. All the information regarding individual persons had been anonymized. 

### 2.2. Data Collection

The following variables of enrolled participants were extracted: sex, date of birth, registration date, residence region, date of diagnosis, diagnosis code, and date of death. 

The target outcome of this study was the emergence of an infectious disease. The disease information of cases was analyzed by healthcare professionals, and it was further organized and standardized during data processing according to the International Classification of Diseases, 10th Revision (ICD-10) code, and the Law of the People’s Republic of China on the Prevention and Treatment of Infectious Diseases [20]. For each participant, the study period was from the date of registration to the date participants turned 18 years, were diagnosed with an infectious disease, died, or the end of the study on 30 June 2017.

According to the main pathogen transmission route, we classified infectious diseases as respiratory, gastrointestinal, mucocutaneous, blood-borne and sexually transmitted, or vector-borne infections. 

### 2.3. Statistical Analysis

Skewed variables were reported as median (interquartile range—IQR). Categorical variables were expressed as frequency (*n*) and proportion (%). The incidence density of infectious disease was defined as the number of new cases divided by the total person-years (PY) over the study period (per 100,000 PY). The incidence of disease was stratified and calculated by gender, age, and region (prefecture-level dimensions; urban or rural; east, south, west, north, and central Shandong Province). Age groups were classified as <1, 1–3, 4–6, 7–9, 10–12, 13–15, and 16–18 years according to age at the registration and diagnosis of participants. Miettinen’s formula was used to compare the incidence density between sex and region [21]. Logarithmic linear regression was conducted to identify the annual incidence trend of infections as follows: Y = α + βx + ε [22]. The annual percentage change (APC) can be inferred from the regression coefficient β. If the APC was significant (*p* < 0.05), the incidence was considered to be increasing or decreasing; otherwise, the incidence was considered stable. The seasonal distribution of infections was described with a radar diagram based on the monthly incidence density. All analyses were conducted using R software (version 4.0.5, R Project for Statistical Computing). Thematic maps were produced by ArcGIS software (version 10.1, ESRI Inc., Redlands, CA, USA). 

## 3. Results

### 3.1. Characteristics of Participants with Infectious Diseases

A total of 891,981 children and adolescents aged 0–18 years were included in the present study. Of the participants, 53.6% were male, and the median age at enrollment was 7 years (IQR: 3–12). More than 570,000 participants were recruited in 2013. More detailed information about the 891,981 participants is presented in Table S1. The distribution of participants across 16 cities of Shandong Province, China, is displayed in Figure S1.

From 2013 to 2018, 18,183 cases of 78 infectious diseases were diagnosed among children, with a median age of 4 years (IQR: 2–9). The total incidence density of infectious diseases was 626.33 per 100,000 PY, with 675.94 per 100,000 PY in males and 568.76 per 100,000 PY in females (*p <* 0.001). Children aged 1–3 years had the highest incidence (1771.97 per 100,000 PY). Children and adolescents in rural regions had a higher incidence than those in urban regions (*p <* 0.001). Among all participants, respiratory infectious diseases were the most common (358.30 per 100,000 PY), while vector-borne infections were the least (2.69 per 100,000 PY). Approximately 50 non-notifiable infectious diseases were reported, constituting 6825 cases (235.09 per 100,000 PY). Only 998 cases of 11 infectious diseases included in the National Immunization Program (NIP) were discovered (Table 1). 

### 3.2. Temporal Distribution of Infectious Diseases

**Figure 1:**
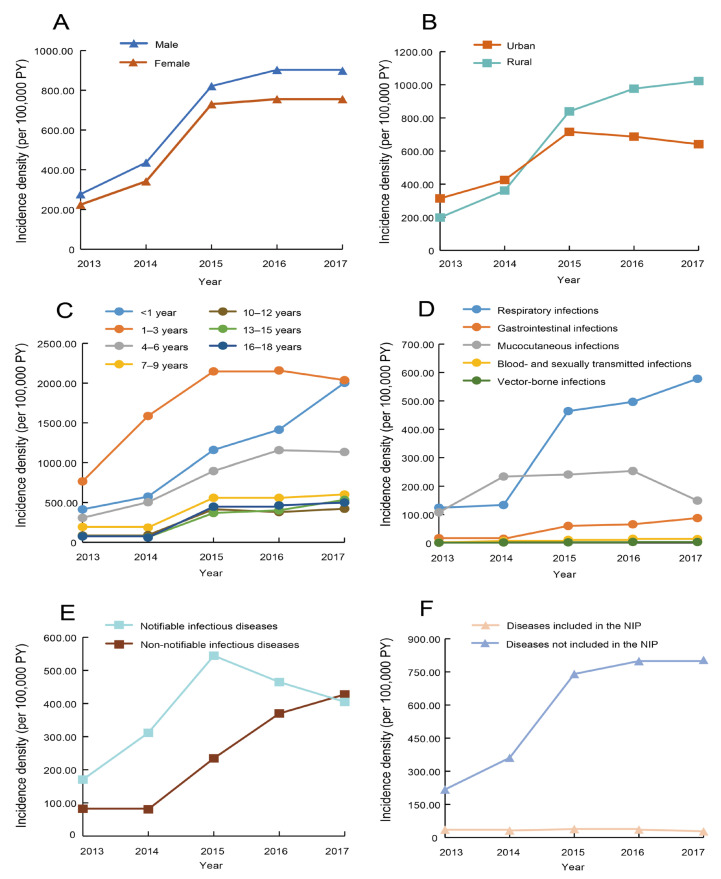
Temporal trends of infectious diseases among children by different subgroups, 2013–2017. (**A**) Sexes. (**B**) Geographical regions. (**C**) Age groups. (**D**) Infectious diseases by transmission route. (**E**) Infectious diseases by reporting type. (**F**) Infectious diseases by whether they are included in the NIP. PY—person-years; NIP—National Immunization Program.

As shown in Table S2, the overall incidence density revealed an increasing trend from 2013 (252.69 per 100,000 PY) to 2017 (832.47 per 100,000 PY), with an APC of 36.9% (*p* = 0.03). The increasing trend was also shown in the subgroup analysis by sex (both *p* < 0.05) (Figure 1A). In 2015, the incidence of infections in rural regions surpassed that of urban regions and had a more pronounced upward trend throughout the period (Figure 1B). Increasing trends were observed in the incidence of infections among children aged < 1 year, 4–6 years, and 13–15 years, with APCs of 50.0%, 41.0%, and 77.2%, respectively (all *p* < 0.05, Figure 1C). Respiratory infections, gastrointestinal infections, and vector-borne infections showed increasing trends (all *p* < 0.05, Figure 1D). It was noted that the incidence density of non-notifiable infectious diseases demonstrated a significant increasing trend (APC = 61.8%, *p* = 0.01) (Figure 1E). Additionally, the incidence of infectious diseases included in the NIP remained stable, while those not included increased significantly (APC = 40.7%, *p* < 0.05) (Figure 1F).

As shown in Table S3 in detail, seven infectious diseases, including pneumonia (APC = 66.9%, *p* = 0.007), scarlet fever (APC = 33.9%, *p* = 0.04), rubella (APC = 85.6%, *p* = 0.008), zoster (APC = 101.5%, *p* = 0.02), molluscum contagiosum (APC = 139.36%, *p* < 0.001), viral conjunctivitis (APC = 79.0%, *p* = 0.03) and syphilis (APC = 26.1%, *p* = 0.04), represented significant increasing trends, while mumps displayed a declining trend (APC = −27.0%, *p <* 0.001). Figure S2 illustrates the seasonal pattern of 41 infectious diseases. In general, respiratory diseases peaked in December and January, and mucocutaneous diseases peaked in June, July, and August. 

### 3.3. Age Distribution of Infectious Diseases

**Figure 2:**
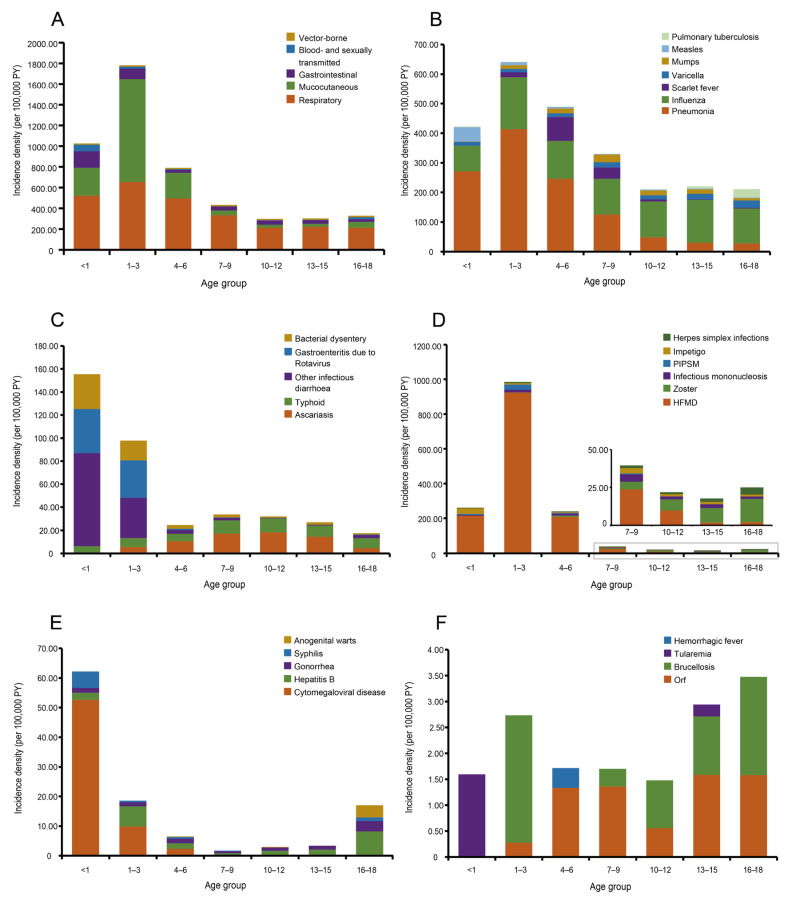
Age distribution of 78 infectious diseases among children in Shandong Province, China. (**A**) Total infections by five categories. (**B**) Respiratory infections. (**C**) Gastrointestinal infections. (**D**) Mucocutaneous infections. (**E**) Blood-borne and sexually transmitted infections. (**F**) Vector-borne infections. PIPSM—picornavirus infections presenting in the skin or mucous membranes; HFMD—hand, foot, and mouth disease; PY—person-years.

**Figure 3:**
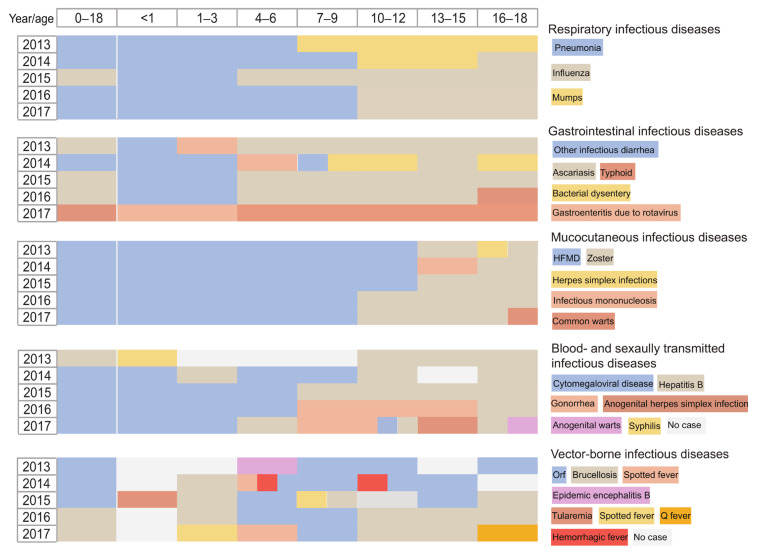
The greatest incidence density of infectious diseases among children and adolescents by different age groups from 2013 to 2017. HFMD—hand, foot, and mouth disease.

Our results showed that children aged 1–3 years had the highest incidence of infections (1771.97 per 100,000 PY), while children aged 10–12 years had the lowest (287.85 per 100,000 PY, Figure 2A). Respiratory diseases were the predominant infections among all age groups, but children aged 1–3 years had the highest incidence. Gastrointestinal, mucocutaneous, and blood-borne and sexually transmitted infections tended to mostly affect children aged < 1 year, 1–3 years, and <1 year, respectively (Figure 2A). Pneumonia was the most common respiratory infection in children aged < 10 years, and among this group, children aged 1–3 years had the highest incidence (Figure 3). Influenza was prevalent in all age groups, especially among adolescents aged 10 years and above. It has replaced mumps as the leading disease in adolescents since 2015. Scarlet fever occurred mostly in children aged 4–9 years, while measles mainly affected children < 1 year (Figure 2B). Ascariasis and typhoid were the most common gastrointestinal diseases in children aged ≥ 4 years, whereas other infectious diarrhea and gastroenteritis due to rotavirus were most prevalent in those aged < 4 years. In 2017, the leading gastrointestinal infection changed from ascariasis to typhoid in children aged ≥ 4 years and from other infectious diarrhea to gastroenteritis due to rotavirus in children aged < 4 years (Figure 2C and Figure 3).

Hand, foot, and mouth disease (HFMD) and zoster were the most common mucocutaneous infections among children aged < 10 years and adolescents ≥ 10 years, respectively. Herpes simplex infections, infectious mononucleosis, and common warts were also major threats in those aged ≥ 10 years (Figure 2D and Figure 3). Among all blood-borne and sexually transmitted infections, cytomegaloviral disease dominated in children aged < 7 years, especially in those aged < 1 year. In children aged ≥ 7 years, gonorrhea and anogenital warts replaced hepatitis B as the leading diseases in 2016 (Figure 2E and Figure 3). More information on the population distribution of the 78 infectious diseases is shown in Table S4.

### 3.4. Spatial Distribution of Infectious Diseases

**Figure 4:**
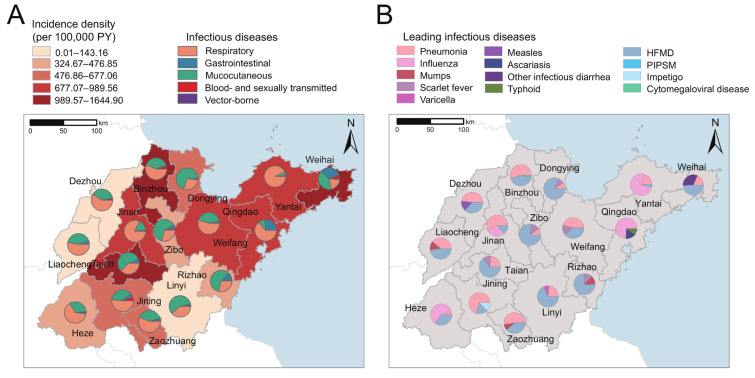
Geographic distribution of infectious diseases in children in Shandong Province, China. (**A**) Geographic distribution of the cumulative incidence and proportions of five categories of infections by transmission route in 16 cities. (**B**) The three diseases with the highest incidence density in each city. PIPSM—picornavirus infections presenting in the skin or mucous membranes; HFMD—hand, foot, and mouth disease; PY—person-years.

As shown in Figure 4 and Figure S3, the central and northeastern coastal cities had a higher incidence density than the southern and western cities of Shandong Province. During the study period, Taian (1644.90 per 100,000 PY), Binzhou (1261.22 per 100,000 PY), and Weihai (989.56 per 100,000 PY) had the highest incidence, while Dezhou had the lowest (143.16 per 100,000 PY, Figure 4A). In most cities, respiratory and mucocutaneous infectious diseases were the most frequent causes of infections. HFMD, pneumonia, and influenza were the three most common diseases in children and adolescents (Figure 4). Gastrointestinal infections, especially ascariasis, typhoid, and other infectious diarrhea, increased significantly in the eastern coastal regions of Shandong Province (Figure 4 and Figure S3). The incidence of blood-borne and sexually transmitted infections was highest in Weihai, Jining, and Binzhou (Figure 4A).

## 4. Discussion

Using the multi-source medical big data collected from 1 January 2013 to 30 June 2017, the epidemiological features of infectious diseases in children and adolescents were analyzed. A total of 18,183 cases of 78 infections were identified among the 891,981 participants aged 0–18 years in Shandong Province, China. In the present study, we provided a complete spectrum of infectious diseases in children and adolescents, involving 50 non-notifiable infectious diseases, which have thus far not attracted particular attention. 

During the study period, the overall incidence of childhood infectious diseases showed a clear upward trend, which may be related to the improvement in the level of diagnosis and treatment, the change in people’s medical habits, the increase in microbial resistance and environmental pollution [19,23]. Notably, respiratory infectious diseases were the most common infections affecting children and experienced a significant increase, while pneumonia and influenza accounted for 78.72% of respiratory cases. The leading cause of death in China was pneumonia, accounting for 17% of deaths in children under 5 years [24]. Previous studies have revealed that risk factors contributing to the high incidence of pneumonia included non-exclusive breastfeeding (in the first 4 months of life), malnutrition, indoor air pollution, crowding, and a lack of immunization [25,26]. Influenza surveillance data obtained from the sentinel hospitals in Shenyang, China, showed that the outbreaks were mainly caused by the spread of emerging and re-emerging viruses originating from homologous influenza viruses [27]. Children aged 0–14 years were much more susceptible to the influenza virus, which was related to a slow build-up of immunity among young populations and school life [28]. Vaccines against Streptococcus pneumonia and influenza are not part of the routine childhood vaccination programs in many countries worldwide, including China [18]; routine childhood immunization is being urgently strengthened to reduce the high burden of pneumonia and influenza in China. 

In recent years, many countries including England and Korea have experienced a strong resurgence in scarlet fever [29,30], which is consistent with our study. One reason for the significant increase in scarlet fever might be antibiotic resistance due to the expansion of S pyogenes from a single clonal lineage to multiclonal lineages [30]. Additionally, some studies attributed the upsurge of scarlet fever in China to the natural cyclical pattern of the disease and the increasing number of susceptible children after the relaxation of the one-child policy [31,32,33]. Zoster is caused by the reactivation of the latent varicella-zoster virus and can occur at any age. It was more common among older people aged ≥ 50 years, especially those with an immunocompromised status and those on immunosuppressant drugs [34]. Zoster in children could be due to having had varicella before 1 year of age, intrauterine varicella exposure, or a history of varicella vaccination [35]. In the present study, zoster was the most common mucocutaneous infection among children and adolescents, followed by HFMD; a significant increasing trend was observed, which has previously been less widely reported. For now, the increasing trend of zoster among children and adolescents cannot be confidently explained and needs to be confirmed by more studies [36]. The incidence of zoster after a natural varicella infection in children is higher than after a varicella vaccination, so routine varicella vaccination is necessary [37]. 

During the study period, 11 diseases included in the NIP of China (epidemic cerebrospinal meningitis, epidemic encephalitis B, hemorrhagic fever, hepatitis A, hepatitis B, measles, mumps, pulmonary tuberculosis, rubella, tetanus, and whooping cough) remained at a stable low level among children and adolescents. Vaccines against hepatitis A, meningitis, encephalitis B and mumps were newly included in the NIP in 2008, and school-age children are regularly vaccinated [38]. With the implementation of population-based vaccination programs, the incidence of these vaccine-preventable diseases has experienced a large decline in China [39]. The mumps-containing vaccine was included in China’s Expanded Program on Immunization in 2008, and the mumps epidemic has been greatly weakened since then [40,41]. Our study found similar results, providing another piece of evidence on the achievements of the NIP. Although rubella was also included in the NIP, its incidence in our study showed a significant increasing trend; infants aged < 1 year had the highest incidence. Previous studies have demonstrated that infants aged 0–8 months were too young for routine immunization. The immunity in newborns was temporary and began to wane at 6 months of life, which made infants the most susceptible to rubella [42,43]. Another study found that women of childbearing age had lower levels of rubella antibodies, increasing the risk of congenital rubella syndrome in newborns to some extent [44,45]. Therefore, improving the vaccination coverage for infants and women of childbearing age could help reduce the incidence of rubella among young children [46]. 

A remarkable feature of the COVID-19 pandemic is that the disease is much more common in adults than in children [47]. This unexplained phenomenon has revived interest in age patterns of infectious disease. In the present study, we identified children who experienced different leading infections at different times of their lives, from infancy through late adolescence due to physiological, morphological, behavioral, and environmental effects [48]. For younger children aged ≤ 6 years, HFMD, pneumonia, measles, other infectious diarrhea, gastroenteritis due to rotavirus, bacterial dysentery, and cytomegaloviral disease were the major infectious diseases; for school-age children aged > 6 years, varicella, pulmonary tuberculosis, ascariasis, typhoid, zoster, and hepatitis B were the leading infections. Furthermore, we found that the leading infectious diseases by age group shifted during the study period, particularly the blood-borne and sexually transmitted diseases. In our study, gonorrhea, anogenital warts, and anogenital herpes simplex infection replaced hepatitis B as the leading infections in 2016 among participants aged ≥ 7 years. An epidemiological study has reported that about half of the 20 million new cases of sexually transmitted infections in the United States were adolescents [49]. An increase in the incidence of HIV/AIDS, together with gonorrhea and syphilis, was noted in a study of students aged 6–22 years, which also suggests that sexually transmitted diseases have become a priority [8]. Effective prevention and control strategies targeting vulnerable age groups should be carried out in public health practice.

The different geographic regions in this study were observed to have different patterns and trends of infectious diseases. The incidence density of infections ranged from 143.16 per 100,000 PY (Dezhou) to 1644.90 per 100,000 PY (Taian). The huge variation in incidence is mainly due to the differences in environmental, social, and economic factors [50]. The central and northeastern coastal cities were identified as the areas where children had the highest risk of contracting infectious diseases. A possible reason is that Jinan, as the capital of Shandong Province, and Taian, Weihai, and other coastal cities, as tourist cities, have attracted a larger floating population [51,52]. Moreover, eastern coastal regions had a higher and increasing incidence of gastrointestinal infections. This might be attributed to the high relative humidity, as well as the high consumption of raw seafood [53,54,55]. Additionally, we found that the incidence of infectious diseases was higher in rural than urban regions, with a more pronounced increasing trend. On the one hand, the increase in China’s left-behind children in rural areas, with poor living and sanitary conditions, provides more opportunities for the spread of infectious diseases among children; on the other hand, low health literacy makes rural residents unable to manage their own health effectively [56,57]. Therefore, measures addressing common infections and the poor socio-economic situation would help reduce these regional inequalities. 

This study gave good estimates of the incidence density and trends of infectious diseases among children and adolescents in Shandong Province, China, using multi-source medical big data. However, three main limitations should be noted. Firstly, bias could be introduced by the diagnostic standards, experimental conditions, and technical levels of different institutions and doctors. Secondly, the 5-year research period is a little short; for instance, the COVID-19 pandemic may change the incidence density of infectious diseases in Shandong Province and the rest of China, as it has done in many other parts of the world, so our study does not necessarily reflect what is currently happening on the ground and may not be the situation in a post-COVID-19 pandemic era. Thirdly, we cannot avoid the under-reporting that results from not seeking treatment after an infection.

## 5. Conclusions

In conclusion, we identified 78 infectious diseases among 891,981 children and adolescents in Shandong Province, China. The challenges and tasks ahead for infectious diseases have been increasing continuously in the era of the relaxed two-child policy and pre-COVID-19 pandemic. The precise and effective prevention of infections with a high incidence and significant upward trends, particularly non-notifiable infectious diseases, should be implemented. Age and regional targeting efforts are also urgently needed.

## Figures and Tables

**Table 1:** Characteristics of participants with infectious diseases.

	Total (*n* = 18,183)	Male (*n* = 10,540)	Female (*n* = 7643)
Age (in years) at diagnosis, median (IQR)	4 (2–9)	4 (2–8)	4 (2–9)
Incidence density of diseases by age group at diagnosis (per 100,000 PY)			
Total (0–18)	626.33	675.94	568.76
<1	1015.66	1144.76	861.28
1–3	1771.97	1915.75	1596.53
4–6	779.44	834.47	713.34
7–9	422.02	428.19	414.76
10–12	287.85	300.21	273.79
13–15	292.49	306.77	276.62
16–18	318.59	350.37	283.80
Incidence density of diseases by geographical region (per 100,000 PY)			
Urban	569.03	608.22	523.35
Rural	680.60	740.34	611.58
Incidence density of diseases by diagnosis year (per 100,000 PY)			
2013	252.69	277.18	224.49
2014	392.45	436.40	341.69
2015	778.96	821.01	730.09
2016	834.79	902.77	755.49
2017	832.47	898.55	755.41
Incidence density of diseases by transmission route (per 100,000 PY)			
Respiratory	358.30	373.95	340.16
Gastrointestinal	48.19	49.00	47.25
Mucocutaneous	207.19	239.08	170.19
Blood-borne and sexually transmitted	9.95	11.03	8.71
Vector-borne	2.69	2.89	2.46
Incidence density of diseases by reporting type (per 100,000 PY)			
Notifiable infectious diseases	391.23	432.18	343.73
Non-notifiable infectious diseases	235.09	243.76	225.03
Incidence density of diseases by whether included in the NIP (per 100,000 PY)			
Infectious diseases included in the NIP	34.38	41.36	26.27
Infectious diseases not included in the NIP	591.95	634.57	542.49

IQR—interquartile range; PY—person-years; NIP—National Immunization Program.

## Data Availability

The data presented in this study are available on request from the corresponding author. The data are not publicly available due to privacy restrictions and intellectual property protection.

## Supplementary Materials

The following supporting information can be downloaded at: https://www.mdpi.com/article/10.3390/children11030309/s1, Figure S1: Sampling procedures and geographical distribution of 891,981 participants in Shandong Province, China, 2013-2017; Table S1: Basic information of study participants; Table S2: Changes on incidence density of infectious diseases by different subgroups, 2013–2017; Table S3: Changes on incidence density of infectious diseases among participants, 2013–2017; Figure S2: Seasonal pattern of infectious diseases; Table S4: Incidence density of 78 infectious diseases among children and adolescents stratified by sex, geographical region, and age group; Figure S3: Incidence density of infections by transmission route in 16 prefecture-level cities of Shandong Province, China.
