# Supplementary material for: Epidemiological Features of Infectious Diseases in Children and Adolescents: A Population-Based Observational Study in Shandong Province, China, 2013–2017
Source: Children (Basel). 2024 Mar 5;11(3):309. doi: 10.3390/children11030309 (PMC10968946; doi:10.3390/children11030309)
Supplement: Supplementary file 1 [file children-11-00309-s001.zip › children-2850466-supplementary.pdf]

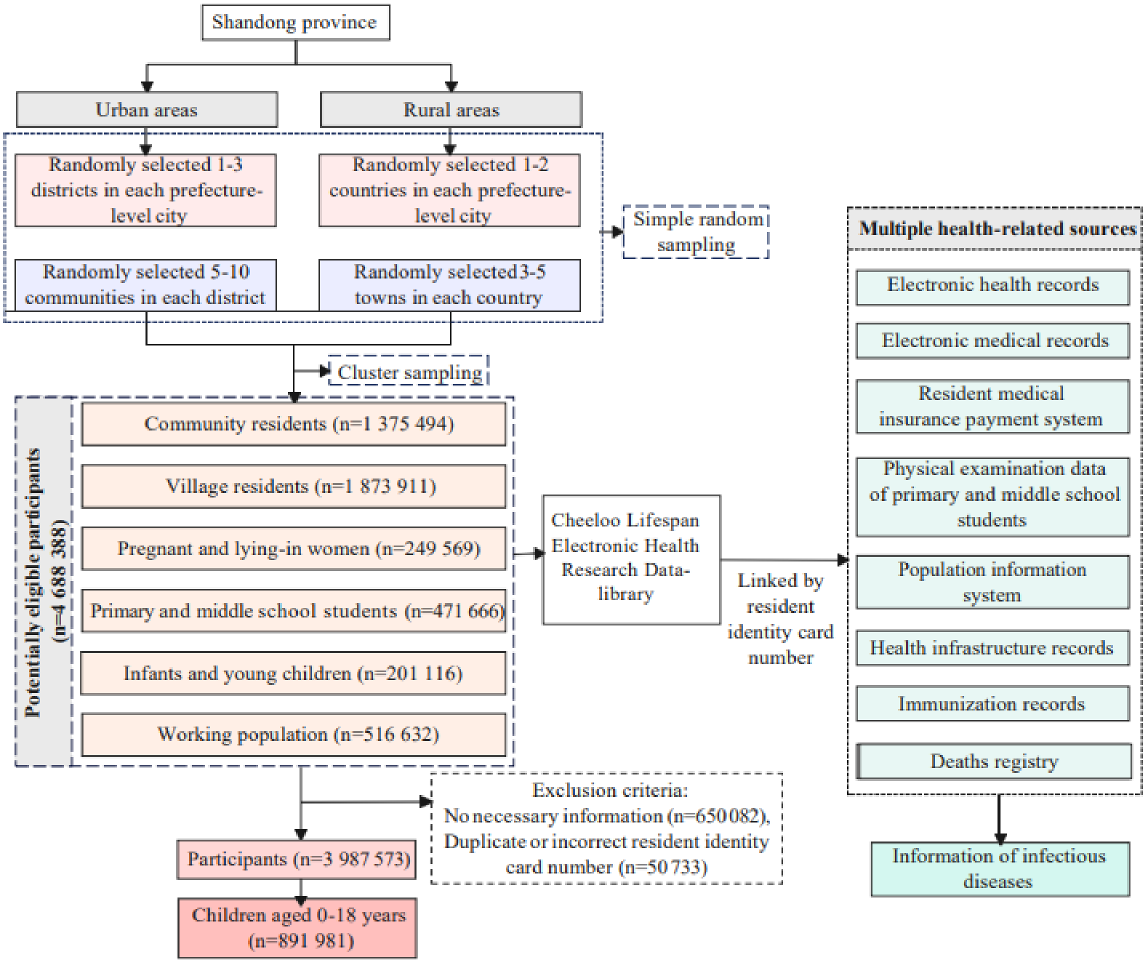

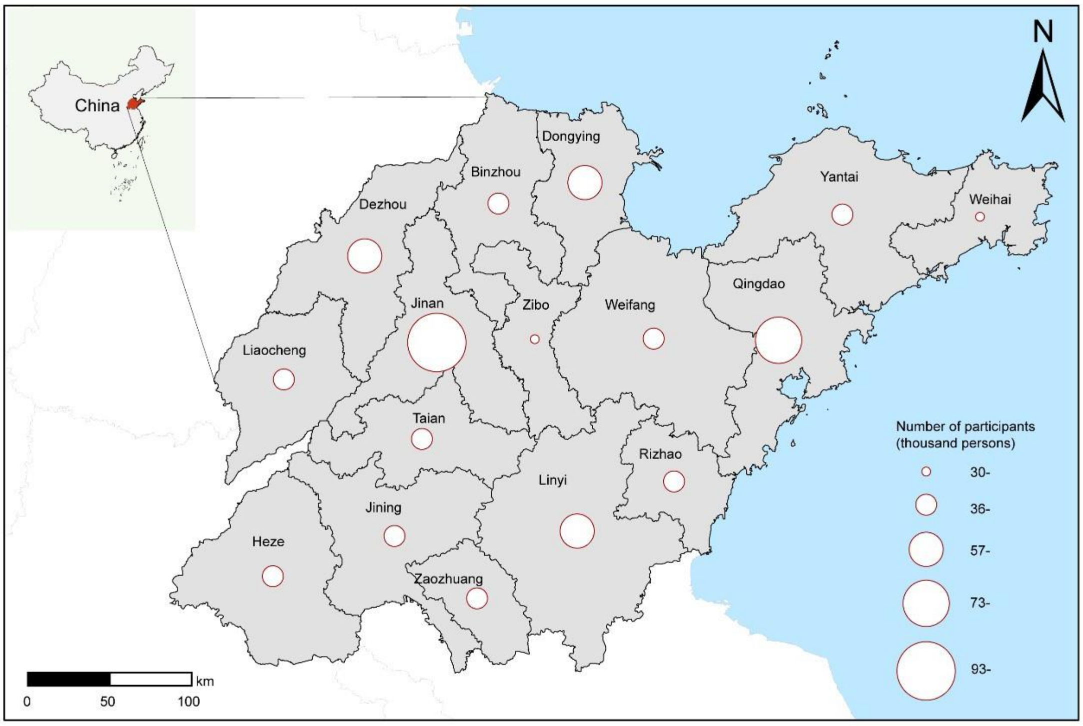

Figure S1: Sampling procedures and integration of the Cheeloo Lifespan Electronic Health Research Data-library

Table S1: Basic information of study participants

	Total (N=891 981)	Male (n=478 437)	Female (n=413 544)
**Age (years) at registration, median (IQR)**	7 (3-12)	7 (3-12)	7 (3-12)
**Age group at registration, n (%)**			
<1	115 349 (12.9)	62 749 (13.1)	52 600 (12.7)
1-3	127 652 (14.3)	70 289 (14.7)	57 363 (13.9)
4-6	168 795 (18.9)	91 599 (19.1)	77 196 (18.7)
7-9	155 385 (17.4)	83 097 (17.4)	72 288 (17.5)
10-12	133 503 (15.0)	70 691 (14.8)	62 812 (15.2)
13-15	105 684 (11.8)	55 441 (11.6)	50 243 (12.1)
16-18	85 613 (9.6)	44 571 (9.3)	41 042 (9.92)
**Geographical region, n (%)**			
Urban	445 544 (50.0)	238 772 (49.9)	206 772 (50.0)
Rural	446 437 (50.0)	239 665 (50.1)	206 772 (50.0)
**Time of registration, n (%)**			
2013	572 106 (64.1)	306 299 (64.0)	265 807 (64.3)
2014	98 210 (11.0)	52 332 (10.9)	45 878 (11.1)
2015	105 865 (11.9)	57 556 (12.0)	48 309 (11.7)
2016	79 505 (8.9)	42 942 (9.0)	36 563 (8.8)
2017	36 295 (4.1)	19 308 (4.0)	16 987 (4.1)
**Residence area, n (%)**			
East	173 703 (19.5)	89 994 (18.8)	83 709 (20.2)
South	273 488 (30.7)	152 581 (31.9)	120 907 (29.2)
West	110 021 (12.3)	60 000 (12.5)	50 021 (12.1)
North	107 486 (12.1)	55 769 (11.7)	51 717 (12.5)
Central	227 283 (25.5)	120 093 (25.1)	107 190 (25.9)

IQR, interquartile range; East area included Yantai, Qingdao, and Weihai; South area included Zaozhuang, Linyi, Rizhao, Heze, and Jining; West area included Dezhou and Liaocheng; North area included Dongying and Binzhou; Central area included Weifang, Zibo, Jinan, and Taian.

Table S2: Changes on incidence density of infectious diseases by different subgroups, 2013-2017

	**Incidence density (per 100 000 PY)**					**APC**	***P***
	**2013**	**2014**	**2015**	**2016**	**2017**	**(%)**	**value**
**Age group (years) at diagnosis**							
Total (0-18)	252.69	392.45	778.96	834.79	832.47	36.9	0.03
<1	413.97	574.83	1159.14	1413.88	2003.16	50.0	0.002
1-3	766.25	1586.37	2147.34	2159.59	2037.13	25.4	0.09
4-6	307.58	504.24	896.11	1156.51	1133.38	41.0	0.02
7-9	193.38	185.30	559.32	558.64	601.52	40.1	0.05
10-12	85.45	90.61	414.90	380.19	421.94	58.9	0.05
13-15	77.12	63.14	368.52	402.56	533.71	77.2	0.04
16-18	74.56	59.62	448.30	463.90	499.98	79.6	0.06
**Sex**							
Male	277.18	436.40	821.01	902.77	898.55	36.1	0.03
Female	224.49	341.69	730.09	755.49	755.41	38.0	0.04
**Geographical region**							
Urban	313.70	425.26	715.81	687.09	640.97	21.0	0.08
Rural	198.63	361.29	839.24	976.60	1022.40	53.3	0.02
**Infectious diseases by transmission route**							
Respiratory	124.31	133.86	463.98	496.58	577.91	55.0	0.03
Gastrointestinal	17.07	14.76	60.20	65.92	87.90	61.2	0.03
Mucocutaneous	108.02	234.21	241.23	253.83	149.38	7.6	0.61
Blood- and sexually transmitted	2.13	7.46	11.03	14.63	13.60	54.9	0.05
Vector-borne	1.16	2.16	2.53	3.83	3.66	33.2	0.02
**Infectious diseases by reporting type**							
Notifiable infectious diseases	170.27	311.67	544.44	464.85	404.98	23.8	0.15
Non-notifiable infectious diseases	82.42	80.78	234.52	369.94	427.48	61.8	0.01
**Infectious diseases by whether included in the NIP**							
Infectious diseases included in the NIP	35.30	31.52	38.44	35.69	27.99	-3.3	0.47
Infectious diseases not included in the NIP	217.40	360.94	740.52	799.09	804.47	40.7	0.03

PY, person-years; APC, annual percentage change; NIP, National Immunization Program.

Table S3: Changes on incidence density of infectious diseases among participants, 2013-2017

Infectious diseases	Incidence density (per 100 000 PY)						APC	***P***
	**2013-17**	**2013**	**2014**	**2015**	**2016**	**2017**	**(%)**	**value**
**Respiratory infectious diseases**								
Pneumonia	150.22	48.48	52.91	132.01	244.80	291.97	66.9	0.007
Influenza*	131.00	20.36	22.06	244.21	163.70	190.72	91.1	0.08
Scarlet fever*	26.32	10.08	22.23	29.50	33.51	35.32	33.9	0.04
Varicella	16.06	9.31	6.47	19.37	17.79	31.15	40.9	0.06
Mumps*†	15.82	28.51	17.92	14.31	10.81	7.59	-27.0	<0.001
Measles*†	5.86	1.94	5.31	7.90	8.62	3.14	15.6	0.55
Pulmonary tuberculosis*†	5.13	2.52	2.32	7.75	6.70	5.49	29.9	0.16
Roseola infantum	2.51	1.94	1.49	3.72	3.28	1.31	0.0	1.00
Rubella*†	2.38	0.58	0.66	2.53	3.01	6.02	85.6	0.008
Whooping cough*†	1.76	0.19	1.82	1.49	2.33	3.14	78.8	0.08
Meningococcal disease	0.45	0.39	0.17	0.45	0.55	0.78	-	-
Influenza A H1N1*	0.31	0.00	0.17	0.30	0.41	0.78	-	-
Legionellosis	0.21	0.00	0.00	0.15	0.41	0.52	-	-
Epidemic cerebrospinal meningitis*†	0.14	0.00	0.17	0.30	0.14	0.00	-	-
Streptococcal pharyngitis	0.07	0.00	0.00	0.00	0.27	0.00	-	-
Aspergillosis	0.03	0.00	0.00	0.00	0.14	0.00	-	-
Blastomycosis	0.03	0.00	0.00	0.00	0.14	0.00	-	-
Cryptococcosis	0.03	0.00	0.17	0.00	0.00	0.00	-	-
**Gastrointestinal infectious diseases**								
Ascariasis	12.19	8.34	0.33	22.50	17.64	7.59	46.0	0.56
Typhoid*	9.47	0.00	0.33	6.26	14.63	32.44	-	-
Other infectious diarrhea*	9.37	1.55	8.63	9.39	11.35	17.27	66.4	0.05
Gastroenteritis due to rotavirus	6.10	4.85	1.00	4.02	6.56	18.57	58.0	0.20
Bacterial dysentery*	5.17	1.36	3.98	8.94	5.61	4.71	32.7	0.24
Intestinal infections due to Escherichia coli	1.86	0.00	0.00	2.68	4.10	1.57	-	-
Enterobiasis	1.45	0.78	0.00	1.64	2.32	2.62	425.1	0.48
Amoebic dysentery*	1.10	0.00	0.17	2.23	1.91	0.52	-	-
Enteritis due to norovirus	0.31	0.00	0.00	1.19	0.14	0.00	-	-
Infections due to other Salmonella	0.24	0.00	0.00	0.00	0.41	1.05	-	-
Hepatitis A*†	0.21	0.00	0.17	0.45	0.27	0.00	-	-
Bacterial foodborne intoxications, unspecified	0.14	0.00	0.00	0.15	0.14	0.52	-	-
Paratyphoid*	0.14	0.00	0.00	0.15	0.14	0.52	-	-
Taeniasis	0.14	0.00	0.00	0.15	0.27	0.26	-	-
Cysticercosis	0.10	0.00	0.00	0.15	0.14	0.26	-	-
Enteritis due to adenovirus	0.10	0.19	0.17	0.00	0.14	0.00	-	-
Capillariasis	0.07	0.00	0.00	0.30	0.00	0.00	-	-
Epidemic myalgia	0.03	0.00	0.00	0.00	0.14	0.00	-	-

**Mucocutaneous infectious diseases**								
Hand, foot, and mouth disease[★]	170.33	101.04	221.77	200.25	191.19	90.26	-3.7	0.82
Zoster	7.03	1.16	1.33	7.45	12.58	12.56	101.5	0.02
Infectious mononucleosis	6.10	2.13	7.80	7.30	6.29	6.28	21.5	0.31
Picornavirus infections presenting in the skin or mucous membranes	5.03	1.16	0.66	5.07	10.39	6.80	87.4	0.08
Impetigo	4.72	0.97	1.00	7.90	7.11	5.49	72.2	0.10
Herpes simplex infections	3.07	0.97	0.50	3.58	5.61	4.19	70.7	0.10
Common warts	3.00	0.00	0.17	1.79	5.74	8.37	-	-
Molluscum contagiosum	1.62	0.19	0.33	0.74	2.32	5.76	139.4	<0.001
Plane warts	1.34	0.00	0.17	1.19	2.87	2.35	-	-
Scabies	1.14	0.00	0.00	0.45	2.74	2.62	-	-
Non-dermatophyte superficial dermatomycoses	1.03	0.00	0.17	0.74	2.87	0.78	-	-
Viral conjunctivitis	0.72	0.19	0.17	0.74	1.23	1.31	79.0	0.03
Trachoma	0.65	0.19	0.17	1.04	0.55	1.57	-	-
Myiasis	0.55	0.00	0.00	0.89	1.23	0.26	-	-
Rabies[★]	0.45	0.00	0.00	1.49	0.27	0.26	-	-
Infantile papular acrodermatitis	0.31	0.00	0.00	0.45	0.55	0.52	-	-
Tetanus[†]	0.07	0.00	0.00	0.15	0.14	0.00	-	-
Echinococcosis[★]	0.03	0.00	0.00	0.00	0.14	0.00	-	-
**Blood- and sexually transmitted infectious diseases**								
Cytomegaloviral disease	4.03	0.00	3.65	4.47	5.06	7.33	-	-
Hepatitis B[★†]	2.89	1.36	2.99	3.58	3.56	2.35	13.6	0.39
Gonorrhea[★]	1.45	0.00	0.17	1.49	3.28	1.83	-	-
Syphilis[★]	0.59	0.39	0.33	0.60	0.82	0.78	26.1	0.04
Anogenital warts	0.52	0.00	0.17	0.30	1.23	0.78	-	-
Hepatitis C[★]	0.14	0.19	0.00	0.30	0.14	0.00	-	-
Trichomoniasis	0.14	0.19	0.17	0.15	0.00	0.26	-	-
Anogenital herpes simplex infection	0.10	0.00	0.00	0.00	0.27	0.26	-	-
Granuloma inguinale	0.03	0.00	0.00	0.00	0.14	0.00	-	-
HIV infection[★]	0.03	0.00	0.00	0.15	0.00	0.00	-	-
Warts of lips or oral cavity	0.03	0.00	0.00	0.00	0.14	0.00	-	-
**Vector-borne infectious diseases**								
Orf	1.07	0.97	1.33	1.04	0.96	1.05	-1.7	0.74
Brucellosis[★]	0.93	0.00	0.17	0.74	2.05	1.57	-	-
Tularemia	0.10	0.00	0.00	0.30	0.00	0.26	-	-
Cat-scratch disease	0.07	0.00	0.00	0.00	0.27	0.00	-	-
Epidemic encephalitis B[★†]	0.07	0.19	0.00	0.00	0.14	0.00	-	-
Erysipeloid	0.07	0.00	0.33	0.00	0.00	0.00	-	-
Glanders	0.07	0.00	0.00	0.15	0.14	0.00	-	-
Hemorrhagic fever[★†]	0.07	0.00	0.17	0.00	0.00	0.26	-	-

Spotted fever	0.07	0.00	0.00	0.15	0.00	0.26	-	-
Typhus fever*	0.07	0.00	0.17	0.15	0.00	0.00	-	-
Dengue*	0.03	0.00	0.00	0.00	0.14	0.00	-	-
Extraintestinal yersiniosis	0.03	0.00	0.00	0.00	0.14	0.00	-	-
Q fever	0.03	0.00	0.00	0.00	0.00	0.26	-	-

PY, person-years; APC, annual percentage change; * Notifiable infectious diseases; † Infectious diseases included in the National Immunization Program; -, not applicable.

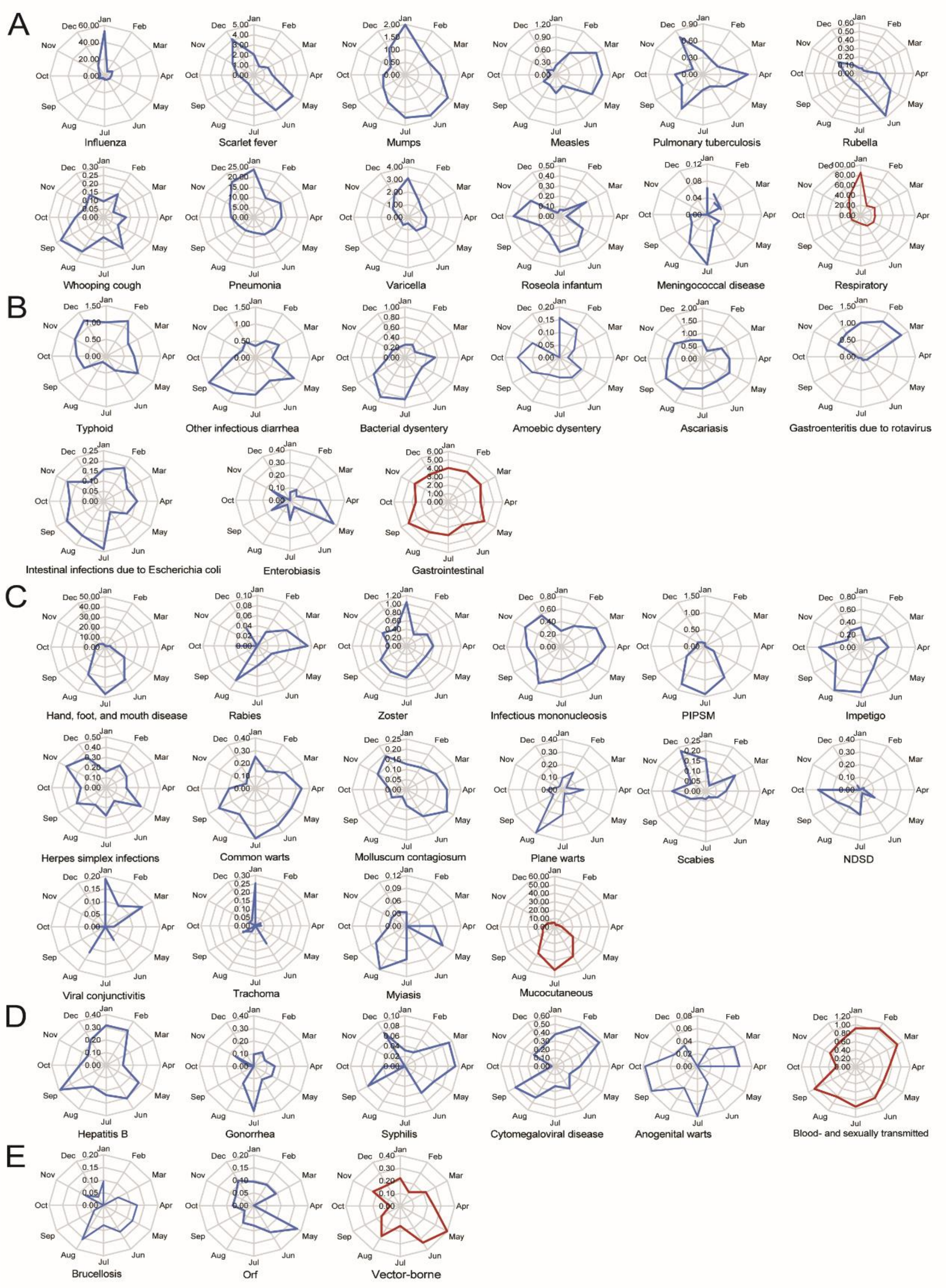

Figure S2: Seasonal pattern of infectious diseases. (A) Respiratory infectious diseases. (B) Gastrointestinal infectious diseases. (C) Mucocutaneous infectious diseases. (D) Blood- and sexually transmitted infectious diseases. (E) Vector-borne infectious diseases. PIPSM, Picornavirus infections presenting in the skin or mucous membranes; NDSD, Non-dermatophyte superficial dermatomycoses.

Table S4: Incidence density of 78 infectious diseases among children and adolescents stratified by sex, geographical region, and age group

Infectious diseases	Cases, n (%)	Incidence density (per 100 000 PY)														Age (years) at diagnosis, median (IQR)
		Total	Male	Female	Ratio (M/F)	Urban	Rural	Ratio (U/R)	<1	1-3	4-6	7-9	10-12	13-15	16-18	
**Respiratory infectious diseases**	10 402 (57.2)	358.30	373.95	340.16	1.10	324.52	390.31	0.83	522.18	654.27	494.85	333.19	212.61	221.69	211.55	6 (3-10)
Pneumonia	4361 (24.0)	150.22	153.91	145.93	1.05	125.33	173.79	0.72	271.06	413.94	246.09	124.90	48.44	30.09	27.47	4 (2-7)
Influenza	3803 (20.9)	131.00	130.25	131.87	0.99	119.53	141.86	0.84	86.10	175.53	127.52	121.33	121.09	145.00	118.41	9 (4-13)
Scarlet fever	764 (4.2)	26.32	30.01	22.03	1.36	30.66	22.20	1.38	0.80	16.95	81.01	38.12	6.84	1.58	2.53	6 (5-7)
Varicella	466 (2.6)	16.06	18.22	13.55	1.34	15.16	16.91	0.90	12.76	11.49	12.58	17.53	14.24	19.01	24.64	10 (6-14)
Mumps	459 (2.5)	15.82	20.98	9.83	2.14	16.01	15.63	1.02	0.80	12.85	16.40	25.54	15.72	14.94	7.58	8 (6-11)
Measles	170 (0.9)	5.86	6.29	5.36	1.17	5.52	6.17	0.90	48.65	9.85	5.34	2.38	2.77	2.26	1.89	1 (0-7)
Pulmonary tuberculosis	149 (0.8)	5.13	6.35	3.72	1.71	5.52	4.76	1.16	2.39	1.09	0.57	0.68	2.03	7.69	28.43	16 (14-18)
Roseola infantum	73 (0.4)	2.51	2.82	2.16	1.31	2.62	2.41	1.09	44.64	4.65	0.00	0.00	0.00	0.00	0.00	0 (0-0)
Rubella	69 (0.4)	2.38	2.31	2.46	0.94	0.50	4.16	0.12	39.06	3.28	0.19	0.34	0.37	0.45	0.32	0 (0-1)
Whooping cough	51 (0.3)	1.76	1.48	2.08	0.71	2.27	1.27	1.78	15.95	2.73	2.29	1.19	0.18	0.23	0.00	1 (0-5)
Meningococcal disease	13 (0.1)	0.45	0.58	0.30	1.94	0.50	0.40	1.23	0.00	0.27	0.57	0.51	0.74	0.45	0.00	8 (6-12)
Influenza A H1N1	9 (0.0)	0.31	0.32	0.30	1.08	0.35	0.27	1.32	0.00	0.55	0.76	0.34	0.18	0.00	0.00	5 (4-9)
Legionellosis	6 (0.0)	0.21	0.19	0.22	0.86	0.35	0.07	5.28	0.00	0.82	0.57	0.00	0.00	0.00	0.00	4 (3-5)
Epidemic cerebrospinal meningitis	4 (0.0)	0.14	0.19	0.07	2.59	0.14	0.13	1.06	0.00	0.00	0.38	0.34	0.00	0.00	0.00	7 (6-8)
Streptococcal pharyngitis	2 (0.0)	0.07	0.00	0.15	0.00	0.07	0.07	1.06	0.00	0.00	0.38	0.00	0.00	0.00	0.00	6 (5-6)
Aspergillosis	1 (0.0)	0.03	0.00	0.07	0.00	0.00	0.07	0.00	0.00	0.00	0.00	0.00	0.00	0.00	0.32	18 (18-18)
Blastomycosis	1 (0.0)	0.03	0.00	0.07	0.00	0.00	0.07	0.00	0.00	0.27	0.00	0.00	0.00	0.00	0.00	2 (2-2)
Cryptococcosis	1 (0.0)	0.03	0.06	0.00	-	0.00	0.07	0.00	0.00	0.00	0.19	0.00	0.00	0.00	0.00	6 (6-6)
**Gastrointestinal infectious diseases**	1399 (7.7)	48.19	49.00	47.25	1.04	33.85	61.78	0.55	158.65	101.98	27.64	38.97	40.49	34.61	25.58	6 (1-11)

Ascariasis	354 (1.9)	12.19	10.90	13.69	0.80	4.60	19.38	0.24	0.00	5.47	10.67	17.19	18.30	14.48	4.42	10 (7-12)
Typhoid	275 (1.5)	9.47	10.07	8.78	1.15	2.12	16.43	0.13	6.38	7.93	6.48	11.40	12.02	9.95	8.84	9 (6-13)
Other infectious diarrhea	272 (1.5)	9.37	10.45	8.11	1.29	11.33	7.51	1.51	80.52	34.72	3.24	2.21	0.37	0.68	2.84	1 (0-2)
Gastroenteritis due to rotavirus	177 (1.0)	6.10	6.16	6.03	1.02	6.16	6.04	1.02	38.27	32.54	1.14	0.34	0.18	0.00	0.32	1 (0-1)
Bacterial dysentery	150 (0.8)	5.17	5.45	4.84	1.13	5.95	4.43	1.34	30.29	17.22	3.05	2.55	1.11	1.81	1.26	2 (0-5)
Intestinal infections due to Escherichia coli	54 (0.3)	1.86	1.67	2.08	0.80	0.99	2.68	0.37	0.00	0.27	0.38	1.02	3.33	3.62	3.47	13 (10-15)
Enterobiasis	42 (0.2)	1.45	1.80	1.04	1.72	0.92	1.95	0.47	0.00	1.09	1.14	1.36	2.77	1.36	0.95	10 (7-12)
Amoebic dysentery	32 (0.2)	1.10	1.22	0.97	1.26	0.71	1.48	0.48	0.80	0.00	0.19	1.70	1.66	1.81	0.95	11 (9-14)
Enteritis due to norovirus	9 (0.0)	0.31	0.19	0.45	0.43	0.07	0.54	0.13	0.00	0.27	0.00	0.34	0.55	0.23	0.63	12 (9-14)
Infections due to other Salmonella	7 (0.0)	0.24	0.26	0.22	1.15	0.00	0.47	0.00	0.00	0.55	0.57	0.17	0.00	0.00	0.32	4 (3-7)
Hepatitis A	6 (0.0)	0.21	0.13	0.30	0.43	0.42	0.00	-	0.00	0.82	0.00	0.17	0.00	0.00	0.63	5 (3-15)
Bacterial foodborne intoxications, unspecified	4 (0.0)	0.14	0.13	0.15	0.86	0.14	0.13	1.06	0.80	0.00	0.19	0.00	0.00	0.00	0.63	10 (3-16)
Paratyphoid	4 (0.0)	0.14	0.19	0.07	2.59	0.21	0.07	3.17	0.00	0.27	0.00	0.00	0.18	0.23	0.32	13 (9-15)
Taeniasis	4 (0.0)	0.14	0.13	0.15	0.86	0.07	0.20	0.35	0.00	0.55	0.00	0.34	0.00	0.00	0.00	6 (3-8)
Cysticercosis	3 (0.0)	0.10	0.00	0.22	0.00	0.00	0.20	0.00	0.00	0.00	0.38	0.17	0.00	0.00	0.00	5 (5-6)
Enteritis due to adenovirus	3 (0.0)	0.10	0.13	0.07	1.72	0.07	0.13	0.53	1.59	0.27	0.00	0.00	0.00	0.00	0.00	0 (0.5-0)
Capillariasis	2 (0.0)	0.07	0.06	0.07	0.86	0.00	0.13	0.00	0.00	0.00	0.00	0.00	0.00	0.45	0.00	14 (14-15)
Epidemic myalgia	1 (0.0)	0.03	0.06	0.00	-	0.07	0.00	-	0.00	0.00	0.19	0.00	0.00	0.00	0.00	5 (5-5)
**Mucocutaneous infectious diseases**	6015 (33.1)	200.16	231.26	164.09	1.41	191.97	207.93	0.92	269.46	992.48	240.94	40.67	22.19	19.23	42.63	2 (1-4)
Hand, foot, and mouth disease	4945 (27.2)	170.33	198.29	137.89	1.44	163.00	177.28	0.92	214.45	923.58	208.54	23.65	9.61	1.58	1.89	2 (1-4)
Zoster	204 (1.1)	7.03	7.82	6.10	1.28	6.30	7.71	0.81	0.00	1.37	7.05	5.11	7.40	9.73	15.47	12 (7-15)
Infectious mononucleosis	177 (1.0)	6.10	6.41	5.73	1.12	8.50	3.82	2.22	4.78	15.31	11.63	4.76	1.85	2.49	1.58	5 (3-7)
Picornavirus infections presenting in the skin or mucous membranes	146 (0.8)	5.03	5.71	4.24	1.35	5.52	4.56	1.21	6.38	30.08	4.38	0.68	0.18	0.00	0.00	2 (1-3)

Impetigo	137 (0.8)	4.72	4.94	4.46	1.11	3.68	5.70	0.65	31.89	7.38	5.72	3.57	1.48	1.58	1.26	4 (0-7)
Herpes simplex infections	89 (0.5)	3.07	2.95	3.20	0.92	2.55	3.55	0.72	3.99	7.11	3.05	1.70	1.29	2.26	4.74	6 (2-14)
Common warts	87 (0.5)	3.00	3.66	2.23	1.64	2.62	3.35	0.78	0.00	1.91	1.72	1.53	1.66	4.07	11.05	14 (8-17)
Molluscum contagiosum	47 (0.3)	1.62	1.60	1.64	0.98	1.49	1.74	0.85	0.00	2.19	2.86	1.70	0.92	0.68	1.89	7 (4-11)
Plane warts	39 (0.2)	1.34	1.54	1.12	1.38	0.71	1.95	0.36	0.00	0.27	0.76	1.36	0.74	0.68	6.00	15 (9-17)
Scabies	33 (0.2)	1.14	1.54	0.67	2.30	0.64	1.61	0.40	0.00	0.27	0.00	0.34	0.37	0.68	7.89	16 (16-17)
Non-dermatophyte superficial dermatomycoses	30 (0.2)	1.03	1.54	0.45	3.45	0.64	1.41	0.45	6.38	0.55	0.19	0.00	0.37	2.04	2.53	13 (1-16)
Viral conjunctivitis	21 (0.1)	0.72	0.58	0.89	0.65	0.50	0.94	0.53	0.00	1.37	0.57	0.17	0.74	0.68	1.58	10 (4-14)
Trachoma	19 (0.1)	0.65	0.71	0.60	1.18	0.42	0.87	0.49	0.80	0.55	0.38	0.34	0.92	0.68	1.26	12 (8-15)
Myiasis	16 (0.1)	0.55	0.71	0.37	1.90	1.06	0.07	15.84	0.00	0.00	0.19	0.17	1.29	1.36	0.32	12 (10-14)
Rabies	13 (0.1)	0.45	0.58	0.30	1.94	0.14	0.74	0.19	0.00	0.55	0.95	0.68	0.18	0.23	0.00	6 (4-8)
Infantile papular acrodermatitis	9 (0.0)	0.31	0.32	0.30	1.08	0.35	0.27	1.32	0.80	1.09	0.00	0.00	0.37	0.23	0.32	3 (2-12)
Tetanus	2 (0.0)	0.07	0.13	0.00	-	0.14	0.00	-	0.00	0.00	0.00	0.00	0.18	0.00	0.32	13 (12-15)
Echinococcosis	1 (0.0)	0.03	0.06	0.00	-	0.00	0.07	0.00	0.00	0.27	0.00	0.00	0.00	0.00	0.00	3 (3-3)
**Blood- and sexually transmitted infectious diseases**	289 (1.6)	9.95	11.03	8.71	1.27	9.35	10.53	0.89	63.78	18.87	6.86	1.70	3.14	3.62	19.26	3 (0-13)
Cytomegaloviral disease	117 (0.6)	4.03	4.23	3.80	1.12	2.41	5.57	0.43	52.64	9.85	2.29	0.17	0.18	0.23	0.00	0 (0-2)
Hepatitis B	84 (0.5)	2.89	3.27	2.46	1.33	3.68	2.15	1.72	2.39	6.84	1.91	0.68	1.48	1.81	8.21	9 (2-16)
Gonorrhea	42 (0.2)	1.45	1.99	0.82	2.43	1.49	1.41	1.06	1.59	1.37	1.52	0.68	1.11	1.36	3.47	11 (4-16)
Syphilis	17 (0.1)	0.59	0.64	0.52	1.23	0.85	0.34	2.53	5.58	0.55	0.57	0.17	0.00	0.00	1.26	2 (0-9)
Anogenital warts	15 (0.1)	0.52	0.45	0.60	0.75	0.57	0.47	1.21	0.00	0.00	0.19	0.00	0.18	0.00	4.10	18 (18-18)
Hepatitis C	4 (0.0)	0.14	0.19	0.07	2.59	0.07	0.20	0.35	0.80	0.00	0.19	0.00	0.18	0.00	0.32	8 (4-13)
Trichomoniasis	4 (0.0)	0.14	0.00	0.30	0.00	0.14	0.13	1.06	0.00	0.00	0.00	0.00	0.00	0.00	1.26	18 (187-18)
Anogenital herpes simplex infection	3 (0.0)	0.10	0.19	0.00	-	0.07	0.13	0.53	0.80	0.00	0.00	0.00	0.00	0.23	0.32	14 (7-15)

Granuloma inguinale	1 (0.0)	0.03	0.00	0.07	0.00	0.00	0.07	0.00	0.00	0.27	0.00	0.00	0.00	0.00	0.00	2 (2-2)
HIV infection	1 (0.0)	0.03	0.06	0.00	-	0.07	0.00	-	0.00	0.00	0.00	0.00	0.00	0.00	0.32	18 (18-18)
Warts of lips or oral cavity	1 (0.0)	0.03	0.00	0.07	0.00	0.00	0.07	0.00	0.00	0.00	0.19	0.00	0.00	0.00	0.00	4 (4-4)
**Vector-borne infectious diseases**	78 (0.4)	2.69	2.89	2.46	1.18	3.04	2.35	1.30	1.59	3.01	2.10	2.38	2.03	3.62	4.10	10 (6-14)
Orf	31 (0.2)	1.07	1.03	1.12	0.92	1.56	0.60	2.58	0.00	0.27	1.33	1.36	0.55	1.58	1.58	9 (7-15)
Brucellosis	27 (0.1)	0.93	1.09	0.74	1.47	1.06	0.80	1.32	0.00	2.46	0.00	0.34	0.92	1.13	1.89	11 (3-15)
Tularemia	3 (0.0)	0.10	0.06	0.15	0.43	0.00	0.20	0.00	1.59	0.00	0.00	0.00	0.00	0.23	0.00	0 (0-8)
Cat-scratch disease	2 (0.0)	0.07	0.06	0.07	0.86	0.00	0.13	0.00	0.00	0.00	0.00	0.17	0.18	0.00	0.00	10 (9-10)
Epidemic encephalitis B	2 (0.0)	0.07	0.13	0.00	-	0.14	0.00	-	0.00	0.00	0.19	0.00	0.00	0.23	0.00	10 (8-11)
Erysipeloid	2 (0.0)	0.07	0.13	0.00	-	0.00	0.13	0.00	0.00	0.00	0.19	0.00	0.18	0.00	0.00	9 (7-10)
Glanders	2 (0.0)	0.07	0.06	0.07	0.86	0.00	0.13	0.00	0.00	0.00	0.00	0.17	0.00	0.23	0.00	10 (9-12)
Hemorrhagic fever	2 (0.0)	0.07	0.13	0.00	-	0.07	0.07	1.06	0.00	0.00	0.38	0.00	0.00	0.00	0.00	6 (6-6)
Spotted fever	2 (0.0)	0.07	0.06	0.07	0.86	0.07	0.07	1.06	0.00	0.27	0.00	0.17	0.00	0.00	0.00	5 (4-6)
Typhus fever	2 (0.0)	0.07	0.06	0.07	0.86	0.14	0.00	-	0.00	0.00	0.00	0.00	0.00	0.23	0.32	16 (14-17)
Dengue	1 (0.0)	0.03	0.00	0.07	0.00	0.00	0.07	0.00	0.00	0.00	0.00	0.00	0.18	0.00	0.00	11 (11-11)
Extraintestinal yersiniosis	1 (0.0)	0.03	0.06	0.00	-	0.00	0.07	0.00	0.00	0.00	0.00	0.17	0.00	0.00	0.00	9 (9-9)
Q fever	1 (0.0)	0.03	0.00	0.07	0.00	0.00	0.07	0.00	0.00	0.00	0.00	0.00	0.00	0.00	0.32	17 (17-17)

PY, person-years; IQR, interquartile range; -, not applicable.

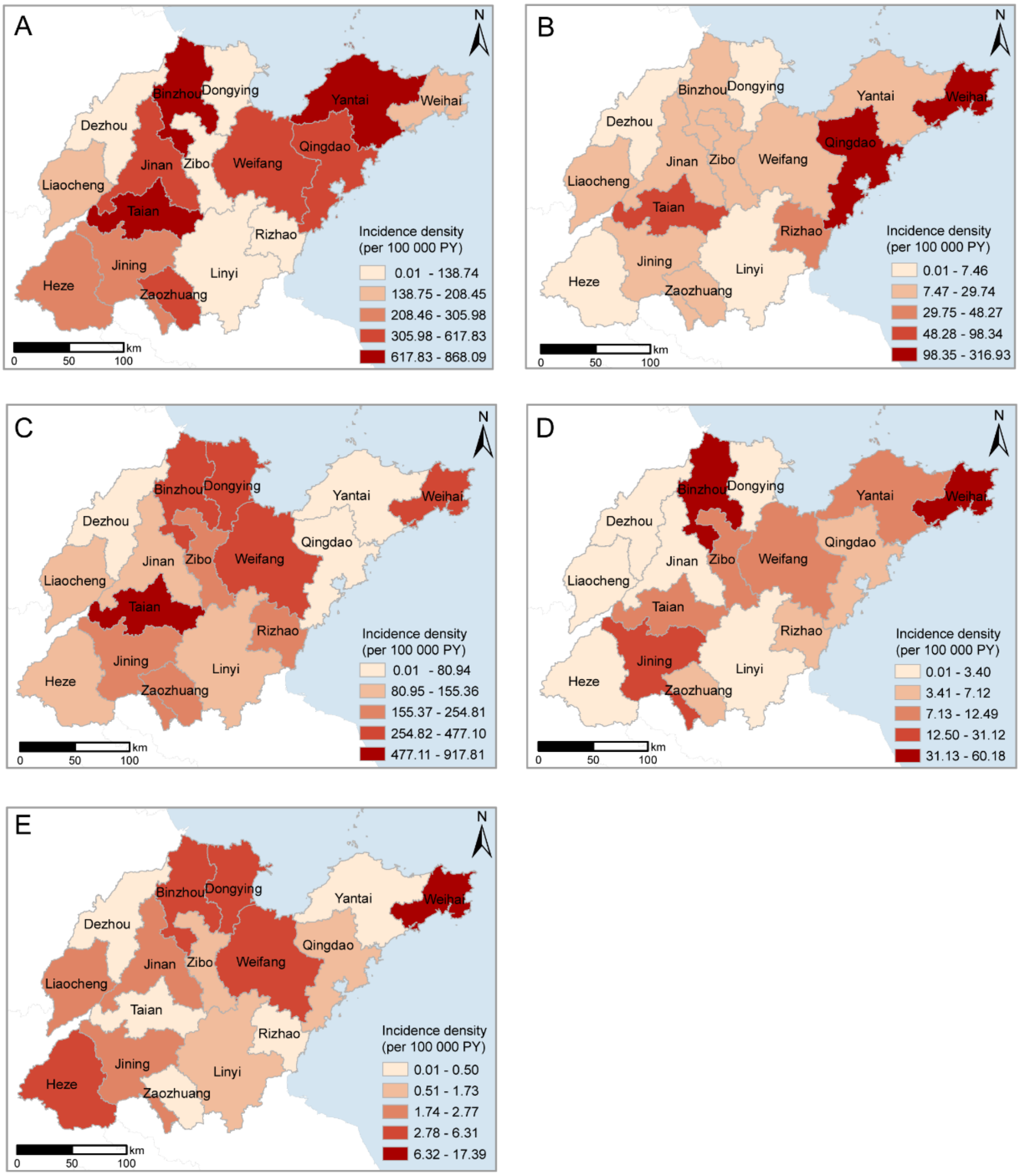

Figure S3: Incidence density of infections by transmission route in 16 prefecture-level cities of Shandong Province, China. (A) Respiratory infectious diseases. (B) Gastrointestinal infectious diseases. (C) Mucocutaneous infectious diseases. (D) Blood- and sexually transmitted infectious diseases. (E)Vector-borne infectious diseases. PY, person-years.
